# Socioeconomic inequity in the utilization of healthcare among people with eating disorders in Australia

**DOI:** 10.1017/S0033291724002290

**Published:** 2024-10

**Authors:** Moin Ahmed, Sarah Maguire, Kelly M. Dann, Francisco Scheneuer, Marcellinus Kim, Jane Miskovic-Wheatley, Danielle Maloney, Natasha Nassar, Michelle Cunich

**Affiliations:** 1 MAINSTREAM The Australian National Centre for Health System Research and Translation, Sydney, NSW, Australia; 2 Boden Initiative, Charles Perkins Centre, Faculty of Medicine and Health (Central Clinical School), The University of Sydney, Sydney NSW 2006, Australia; 3 Faculty of Medicine and Health, InsideOut Institute for Eating Disorders, The University of Sydney and Sydney Local Health District, Sydney NSW 2006, Australia; 4 Sydney Local Health District, Sydney NSW 2050, Australia; 5 Charles Perkins Centre, The University of Sydney, Sydney, NSW 2060, Australia; 6 Child Population and Translational Health Research, Faculty of Medicine and Health, The University of Sydney, Sydney, NSW 2060, Australia; 7 Cardiovascular Initiative, Faculty of Medicine and Health, The University of Sydney, Sydney NSW 2006, Australia; 8 Sydney Institute for Women, Children and their Families, Sydney Local Health District, Sydney NSW 2050, Australia

**Keywords:** access, community healthcare, decomposition analysis, eating disorders, hospitalizations, inequality, socioeconomic inequity

## Abstract

**Background:**

Little is known about socioeconomic equity in access to healthcare among people with eating disorders in Australia. This study aims to measure the extent of inequity in eating disorder-related healthcare utilization, analyze trends, and explore the sources of inequalities using New South Wales (NSW) administrative linked health data for 2005 to 2020.

**Methods:**

Socioeconomic inequities were measured using concentration index approach, and decomposition analysis was conducted to explain the factors accounting for inequality. Healthcare utilization included: public inpatient admissions, private inpatient admissions, visits to public mental health outpatient clinics and emergency department visits, with three different measures (probability of visit, total and conditional number of visits) for each outcome.

**Results:**

Private hospital admissions due to eating disorders were concentrated among individuals from higher socioeconomic status (SES) from 2005 to 2020. There was no significant inequity in the probability of public hospital admissions for the same period. Public outpatient visits were utilized more by people from lower SES from 2008 to 2020. Emergency department visits were equitable, but more utilized by those from lower SES in 2020.

**Conclusions:**

Public hospital and emergency department services were equitably used by people with eating disorders in NSW, but individuals from high SES were more likely to be admitted to private hospitals for eating disorder care. Use of public hospital outpatient services was higher for those from lower SES. These findings can assist policymakers in understanding the equity of the healthcare system and developing programs to improve fairness in eating disorder-related healthcare in NSW.

## Background

Eating disorders affect over one million individuals in Australia (4% of the population) at any given time, with 9% of the Australian population experiencing an eating disorder in their lifetime (Butterfly Foundation, 2022). Eating disorders are associated with psychiatric and medical comorbidities, including anxiety, mood disorders, substance-use and post-traumatic stress disorders, and medical comorbidities across the neuroendocrine, skeletal, nutritional, gastrointestinal, dental, and reproductive systems (Butterfly Foundation, 2018; Hambleton et al., 2022). On average, only half of the people with an eating disorder make a full recovery (Rebecca & Dasha, 2016), and mortality rates are elevated (Weigel, Löwe, & Kohlmann, 2019). Eating disorders impose high economic costs from health system and societal perspectives (Paxton et al., 2012; Tannous et al., 2021), with an estimated cost of AUD 66.9 billion in 2023 (Butterfly Foundation, 2024). Girls and women, those from younger age groups, and First Nations people have a greater risk of experiencing eating disorders (Hay, Girosi, & Mond, 2015). Identifying specific groups who do not have equitable access or utilization of healthcare is crucial to ensuring fair and equal access to treatments, overcoming disparities, and promoting prevention and early intervention (Ndugga, 2024).

Distributional efficiency or ‘equal healthcare for equal need’ is the policy goal of the Australian health system (Pulok, van Gool, & Hall, 2020b). Although health equality – or good health for all – is a goal to strive for, there are unavoidable reasons why the health of people in different groups cannot always be equal, e.g. younger *v.* older age groups. However, there are also unfair and avoidable differences that contribute to health inequality, these are termed *health inequities*. Health inequities are systematic social factors that contribute to the differences in health status between groups, e.g. lack of access to healthcare in remote areas (Morris, Sutton, & Gravelle, 2005).

There is broad consensus that the provision of healthcare services should be according to need (Fleurbaey & Schokkaert, 2011; Wagstaff & van Doorslaer, 2000). The principle that individuals with similar needs for treatment and support should receive equal access to appropriate care, regardless of factors such as socioeconomic status (SES), ethnicity, or geographic location, is called *horizontal equity.* In health equity research, horizontal equity is calculated by identifying *needs variables* – factors which are expected to have an effect on the use of healthcare services (Van de Poel, Van Doorslaer, & O'Donnell, 2012; van Doorslaer & van Ourti, 2011), such as age, and *non-needs variables* ***–*** factors which should not have an effect on healthcare utilization (Fleurbaey & Schokkaert, 2011; Morris et al., 2005; Sözmen & Ünal, 2016; O'Donnell, Van Doorslaer, Wagstaff & Lindelow, 2007), such as income or area of residence. After controlling for need variables, the difference in healthcare utilization between groups is a measure of horizontal inequity. Consistent with the majority of health equity research, the current study focuses on horizontal equity in the context of eating disorder-related healthcare use.

There is a paucity of research investigating the magnitude and trend of inequity in eating disorder-related healthcare utilization. However, previous studies have suggested that lower education levels, working fewer than full-time hours and lower SES are associated with a higher risk of an eating disorder (Burke et al., 2022; Hay et al., 2015, 2017; Mitchison et al., 2020; Mulders-Jones, Mitchison, Girosi, & Hay, 2017). Research also suggests there is inequity in access to care for eating disorders for people living in regional and rural areas of Australia (Hambleton et al., 2020). Despite universal health insurance in Australia, ensuring equity in the provision of healthcare services at all levels, including higher-level specialist care, is a continuing challenge (Dalziel, Huang, Hiscock, & Clarke, 2018; Harris, 2012). Individuals with eating disorders may delay accessing medical treatment (Striegel Weissman & Rosselli, 2017), and only a minority of individuals have their eating disorder detected within primary healthcare or general practice settings (Hoek, 2006; Linville, Brown, & O'Neil, 2012; Mangweth-Matzek & Hoek, 2017). Eating disorders are consequently too often addressed using a late-stage intervention method (Butterfly Foundation, 2015), requiring emergency department presentations and hospital admissions.

Whether Australia's health system is achieving equity in healthcare utilization among people with eating disorders is unclear because they are typically included in general mental health studies, and analyses by diagnostic category are not available (Hashmi, Alam, & Gow, 2020; Hashmi, Alam, Gow, Alam, & March, 2023; Huryk, Drury, & Loeb, 2021). To assess the distributional efficiency or equity performance of the healthcare system, empirical evidence of healthcare utilization at all levels, including community care, emergency department presentations and hospital admissions, is needed (Pulok, van Gool, & Hall, 2020a). Individual state or territory governments also have different aims, timing and types of health policies implemented within their jurisdiction (Maguire & Maloney, 2021; Department of Health, State Government Victoria, 2014), and therefore state-level analysis of health inequities is critical. New South Wales (NSW) is the most populous state in Australia, and 1 in 3 people with an eating disorder in Australia reside in NSW (Morris, Elliott, & Madden, 2022). NSW is characterized by a varied geography, which encompasses urban centers and vast rural areas (Australian Bureau of Statistics, 2022). The majority of NSW residents reside in Greater Sydney, accounting for 61.7% of the population, while the remaining 22% live in country NSW, and 16% live in regional metropolitan areas (Angus, 2020).

The current study aims to assess the horizontal inequity in hospital-related care utilization among people with eating disorders in NSW between 2005 and 2020. Specifically, this research analyses temporal trends in inequities, compares horizontal inequities by public and private hospital settings, and explores the contribution of different need and non-need factors to explaining any inequalities that are observed. This knowledge may help to guide efforts to reduce inequities in specific areas of healthcare and, ultimately, improve the health outcomes of patients with eating disorders.

## An overview of the Australian healthcare system

Australia's healthcare system serves as a compelling case study for examining equity issues. The country's healthcare system operates under a blend of public and private services (Duckett & Willcox, 2015). Medicare, a universal health insurance scheme funded by the Commonwealth Government, provides free-of-charge medical care in public hospitals and subsidized access to private health services in out-of-hospital settings (Duckett & Willcox, 2015). The private sector provides choices for hospitals and specialists (Australian Government PrivateHealth.gov.au, n.d.) and is supported by government policies that encourage private health insurance through financial incentives (Australian Taxation Office, 2024). Medical practitioners set their own fees for primary care and specialist services in a fee-for-service system (Johar, Mu, Van Gool, & Wong, 2017). Patients receive fixed rebates from Medicare for physician charges and contribute a co-payment (Duckett & Willcox, 2015). However, the current system does not allow private health insurance to cover this co-payment for out-of-hospital services (Van Gool, Savage, Viney, Haas, & Anderson, 2009; Wong & Hall, 2018; Wong, Greene, Dolja-Gore, & van Gool, 2017). To minimize out-of-pocket (OOP) costs, additional coverage for OOP payments is available for eligible individuals or families (eligible elderly, low-income, or individuals/families on government allowances) (Van Gool et al., 2009; Wong et al., 2017; Wong & Hall, 2018).

## Data and methods

### Data

Data used in this study consists of linked administrative data of hospital admissions, emergency department visits, mental health ambulatory, and mortality data for NSW. All inpatient separations from public hospitals, public psychiatric hospitals, multi-purpose services, private hospitals, and private day procedure centers in NSW are recorded in the NSW Admitted Patient Data Collection (APDC), whereas information on emergency visits to public hospitals in NSW is recorded in the NSW Emergency Department Data Collection (EDDC). The Mental Health Ambulatory Minimum Data Set (MH-MDS) contains information on the assessment, treatment, rehabilitation, and care of non-admitted patients in the public healthcare setting. The MH-MDS also includes data on hospital-based consultation-liaison services to admitted patients in non-psychiatric and hospital emergency settings, care provided by community workers to admitted patients in staffed community residential settings and mental health promotion and prevention services. Mortality data were obtained from linked data of the Australian Coordinating Registry (ACR) for the Cause of Death Unit Record File (COD URF) and the Registrar of Births, Deaths, and Marriages (RBDM). In NSW, diagnoses are recorded with International Classification of Diseases (ICD)-10 Australian modification (AM) codes in hospital and mental health ambulatory data (Centre for Health Record Linkage (CHeReL), 2023a), whereas diagnoses in the emergency departments are recorded with ICD-10 AM, ICD-9 clinical modification (CM) and Systematized Nomenclature of Medicine Clinical Terms (SNOMED CT) (Centre for Health Record Linkage (CHeReL), 2023b). The NSW APDC has information on up to 55 diagnoses along with the primary diagnosis, whereas NSW EDDC and MHAMB data record only one diagnosis. All these administrative health data were linked by the Centre for Health Record Linkage (CHeReL), and the study team was provided with de-identified data.

### Study sample

In this study, patients with an eating disorder were identified from health administrative and mortality data using eating disorder-related ICD-10 AM, ICD-9 CM and SNOMED CT diagnostic codes, either in the primary or secondary field (online Supplementary Table S1). Our study sample consists of individuals with eating disorders who have utilized healthcare services from hospitals (private or public), emergency departments (public), or public outpatient services in a community setting, or individuals with eating disorders who did not utilize any of the above health services for a given year (mortality data). The final analytical sample included individuals with an eating disorder diagnosis (primary or secondary) from 1 January to 31 December in 2005, 2008, 2011, 2014, and 2017 and from 1 January to 30 June 2020 (as data for private hospitals were provided only for these 6 months in 2020).

### Outcome variables

The outcome variable in this study is healthcare services utilization due to eating disorders. Healthcare utilization is defined as the use of healthcare services (Chiu-Lan, Li-Ting, Ming-kung, Wu-Chien, & Chin-Bin, 2019). Four types of healthcare were considered: public inpatient admissions, private inpatient admissions, emergency department visits, and outpatient days in the public healthcare setting. The hospital admissions due to eating disorders were identified from primary diagnosis only using ICD-10 AM codes (Australian Institute of Health and Welfare, 2018). Emergency department visits due to eating disorders were determined using ICD-10 AM, ICD-9 CM or SNOMED CT codes. Outpatient visits were identified from ICD-10 AM diagnosis codes available in MH-MDS. Three different measures of each outcome variable were constructed for this inequality analysis. Firstly, a dichotomous variable was created, which took a value of one if the individual utilized eating disorder-related healthcare services in a hospital setting (public inpatient admission, private inpatient admission, emergency department visit, or outpatient visit) or zero otherwise. Secondly, the total number of admissions/visits in a hospital setting was used to measure the intensity of healthcare service utilization, which included zero and positive admissions/visits in a given year. Thirdly, a conditional or subsequent number of admissions/visits based on at least one admission/visit was analyzed. Further details of the outcome variables are provided in online Supplementary Table S2.

### Need and non-need variables

Need variables in the current study were age, sex, duration of eating disorder and eating disorder-related common psychiatric and medical comorbidities consistent with recent research (Austin et al., 2021; Hambleton et al., 2022; National Eating Disorder Collaboration, 2023; Treasure & Russell, 2011). SES, marital status, country of birth, and remoteness of residence were non-need factors in this research. Full details for common comorbidities and categorization of need and non-need variables are provided in online Supplementary Tables S3 and S4.

### Socioeconomic status

SES was measured using the Socioeconomic Indexes for Areas (SEIFA) - Index of Relative Socioeconomic Advantage and Disadvantage (IRSAD), consistent with recent studies (Islam, Ormsby, Kabir, & Khanam, 2021; Meadows, Enticott, Inder, Russell, & Gurr, 2015; Mitchison et al., 2020; Siegel, Mielck, & Maier, 2015). SEIFA scores are categorized by decile, with the lowest decile (1) representing the most disadvantaged and the highest (10) representing the least disadvantaged. Details of SEIFA are provided in the appendix.

### Empirical methodology

This study's empirical analysis utilized the concentration index (CI) approach, a widely accepted concept in health inequality research (McGrail, van Doorslaer, Ross, & Sanmartin, 2009; Pulok, van Gool, Hajizadeh, Allin, & Hall, 2020; van Doorslaer et al., 2000), to quantify the health inequalities and horizontal inequities. Socioeconomic health equality refers to equal treatment of individuals from different SES irrespective of needs, whereas the horizontal equity principle pertains to the equal treatment of individuals with similar needs irrespective of socioeconomic factors such as income, area of residence, education, and ethnicity (Culyer & Wagstaff, 1993). Since variation in healthcare use due to differences in need factors such as age, sex, and presence of comorbidities is unavoidable, to measure the inequity correctly, the need factors of individuals should be controlled for (Van de Poel et al., 2012; van Doorslaer & van Ourti, 2011). The horizontal inequity (HI) index is a measure of inequity that is obtained after estimating CI. More details on the estimation of CI and HI are provided in the appendix. HI indices range from −1 to +1, with zero indicating no inequity in healthcare use. A negative and statistically significant HI suggests pro-poor inequity, meaning that healthcare use is more concentrated among people with lower SES when everyone has the same level of need for healthcare. On the contrary, a positive and statistically significant HI suggests pro-rich inequity, meaning that healthcare use is more concentrated among people with higher SES when everyone has the same level of need for healthcare. We refer to the terms ‘pro-rich’ or ‘pro-poor’ based on SEIFA, which are derived using information about the economic and social conditions of people and households within a geographical area.

## Results

### Characteristics of the study sample

Descriptive statistics of the study sample are presented in Table 1 and online Supplementary Table S5. Table 1 shows that most (90%) of the study sample were girls and women, and more than half were aged 15–24 years across the study period. Most individuals in the sample were born in Australia (~90%) and were never married (~80%). Three-quarters of the annual sample were residents of major cities, and approximately 40% were distributed in the first five deciles of SEIFA (relatively more disadvantaged; see online Supplementary Fig. S1). The majority of the study sample had a duration of illness less than one year. Nearly 60% of the sample had a psychiatric or medical comorbidity.

**Table 1.** Summary characteristics of the sample population with eating disorders

	Mean					
	2005 (*n* = 1291)	2008 (*n* = 1374)	2011 (*n* = 1715)	2014 (*n* = 2222)	2017 (*n* = 2051)	2020 (n = 1744)
Type of Eating Disorders						
AN only	0.270	0.253	0.226	0.326	0.373	0.447
BN only	0.120	0.082	0.081	0.121	0.107	0.073
Other or unspecified eating disorders	0.513	0.550	0.552	0.419	0.342	0.353
AN and BN	0.014	0.014	0.012	0.018	0.028	0.027
AN and Other or unspecified eating disorders	0.060	0.055	0.085	0.078	0.112	0.085
BN and Other or unspecified eating disorders	0.018	0.031	0.031	0.029	0.021	0.009
AN and BN and Other or unspecified eating disorders	0.005	0.014	0.014	0.009	0.017	0.006
Need Variable						
Female	0.892	0.884	0.898	0.917	0.906	0.907
Age						
less than 15 years	0.088	0.095	0.092	0.087	0.094	0.101
15–24 years	0.522	0.493	0.502	0.542	0.517	0.544
25–34 years	0.175	0.177	0.169	0.176	0.164	0.173
35–44 years	0.093	0.112	0.111	0.090	0.096	0.080
45 years and above	0.122	0.124	0.127	0.105	0.129	0.103
Comorbidities						
No common psychiatric or medical comorbidity	0.318	0.360	0.391	0.363	0.325	0.381
One or more common psychiatric comorbidity	0.542	0.515	0.483	0.476	0.446	0.461
One or more common medical comorbidity	0.047	0.045	0.043	0.042	0.039	0.037
One or more common psychiatric and medical comorbidity	0.092	0.081	0.083	0.119	0.191	0.120
Duration of any eating disorder						
less than 1 year	0.626	0.582	0.552	0.523	0.522	0.446
1–3 years	0.289	0.245	0.250	0.262	0.259	0.332
over 3 years	0.085	0.174	0.198	0.215	0.220	0.223
Non-need variables						
Australia born^	0.904	0.912	0.911	0.907	0.900	0.907
Country of birth+						
Australia	0.904	0.912	0.911	0.907	0.900	0.907
Europe	0.029	0.023	0.028	0.028	0.036	0.031
Asia	0.029	0.026	0.027	0.024	0.031	0.029
Africa	0.006	0.012	0.008	0.010	0.006	0.005
Oceania, New Zealand, the Americas or Other	0.032	0.027	0.026	0.032	0.026	0.028
Marital status						
Never married	0.770	0.792	0.788	0.819	0.807	0.850
Married or de-facto	0.136	0.140	0.145	0.127	0.131	0.109
Widowed/divorced/separated	0.094	0.068	0.066	0.055	0.062	0.041
Remoteness						
Major city	0.757	0.706	0.759	0.775	0.779	0.754
Regional or remote	0.243	0.294	0.241	0.225	0.221	0.246
Socioeconomic status (SEIFA - IRSAD)						
Decile 1	0.063	0.058	0.047	0.044	0.049	0.040
Decile 2	0.075	0.056	0.066	0.062	0.056	0.056
Decile 3	0.054	0.061	0.058	0.052	0.049	0.055
Decile 4	0.080	0.121	0.086	0.113	0.097	0.117
Decile 5	0.096	0.103	0.083	0.096	0.104	0.113
Decile 6	0.105	0.116	0.118	0.122	0.131	0.147
Decile 7	0.069	0.077	0.090	0.076	0.077	0.086
Decile 8	0.138	0.094	0.112	0.119	0.136	0.105
Decile 9	0.165	0.162	0.171	0.161	0.153	0.150
Decile 10	0.154	0.153	0.169	0.156	0.148	0.133

*Notes*: AN: Anorexia Nervosa; BN: Bulimia Nervosa; ^dichotomous independent variable used for country of birth with 1 ‘born in Australia’ and 0 otherwise in regression analyses; ^+^The classification was based on Cheah, Jackson, Touyz, & Hay, 2020 (Cheah et al., 2020).

### Inequity in healthcare utilization in emergency departments and outpatient visits

CIs for emergency department presentations for eating disorders were not statistically significant, suggesting that they remained equitable from 2005 to 2017, with the exception of 2020, where there was pro-poor inequity as demonstrated by a statistically significant negative horizontal inequity (HI: −0.042 and −0.089; Table 2). Similarly, individuals of more disadvantaged SES were more likely and more frequently to utilize outpatient service during 2008–2020, as demonstrated by statistically significant negative HIs, indicating a pro-poor inequity (Table 2).

**Table 2.** Inequality and inequity indices of healthcare utilization due to eating disorders

Type of healthcare utilization		Inequality (CI, 95% confidence interval)											
		2005		2008		2011		2014		2017		2020	
	Type of visit	CI	95%Con.I	CI	95%Con.I	CI	95%Con.I	CI	95%Con.I	CI	95%Con.I	CI	95%Con.I
Hospital admission	Probability of visit	**0.108**	(0.047–0.168)	**0.261**	(0.203–0.319)	**0.185**	(0.133–0.237)	**0.190**	(0.147–0.234)	**0.117**	(0.071–0.164)	**0.150**	(0.099–0.200)
	Total number of visits	**0.315**	(0.192–0.438)	**0.424**	(0.322–0.525)	**0.365**	(0.266–0.464)	**0.272**	(0.173–0.370)	**0.255**	(0.155–0.356)	**0.282**	(0.172–0.392)
	Conditional number of visits	**0.226**	(0.112–0.339)	**0.254**	(0.161–0.347)	**0.230**	(0.138–0.322)	**0.096**	(0.005–0.187)	**0.159**	(0.066–0.252)	**0.153**	(0.050–0.255)
Emergency department visit	Probability of visit	−0.016	(−0.047 to 0.015)	0.001	(−0.028 to 0.030)	0.010	(−0.024 to 0.044)	0.004	(−0.026 to 0.034)	0.014	(−0.022 to 0.050)	**−0.042**	(−0.082 to −0.002)
	Total number of visits	−0.047	(−0.189 to 0.095)	0.043	(−0.127 to 0.212)	−0.001	(−0.087 to 0.086)	−0.012	(−0.091 to 0.067)	0.032	(−0.034 to 0.099)	**−0.089**	(−0.159 to −0.019)
	Conditional number of visits	0.018	(−0.052 to 0.088)	0.038	(−0.079 to 0.155)	−0.025	(−0.056 to 0.007)	−0.022	(−0.061 to 0.017)	0.009	(−0.021 to 0.039)	−0.021	(−0.052 to 0.010)
Outpatient visit	Probability of visit	−0.049	(−0.113 to 0.015)	**−0.132**	(−0.193 to −0.071)	**−0.223**	(−0.277 to −0.169)	**−0.156**	(−0.203 to −0.109)	**−0.075**	(−0.125 to −0.025)	**−0.236**	(−0.289 to −0.183)
	Total number of visits	−0.055	(−0.133 to 0.023)	−0.002	(−0.076 to 0.072)	**−0.123**	(−0.183 to −0.064)	**−0.084**	(−0.132 to −0.036)	−0.036	(−0.086 to 0.015)	**−0.109**	(−0.160 to −0.058)
	Conditional number of visits	−0.029	(−0.099 to 0.041)	**0.071**	(0.005–0.138)	−0.015	(−0.068 to 0.039)	−0.018	(−0.062 to 0.026)	−0.002	(−0.046 to 0.043)	−0.005	(−0.051 to 0.040)
		**Inequity (HI, 95% confidence interval)**											
Hospital admission	Probability of visit	**0.126**	(0.056–0.196)	**0.254**	(0.198–0.310)	**0.188**	(0.135–0.241)	**0.184**	(0.141–0.227)	**0.114**	(0.068–0.160)	**0.099**	(0.066–0.132)
	Total number of visits	**0.322**	(0.201–0.444)	**0.394**	(0.294–0.495)	**0.342**	(0.244–0.439)	**0.240**	(0.143–0.337)	**0.203**	(0.105–0.302)	**0.247**	(0.139–0.356)
	Conditional number of visits	**0.219**	(0.107–0.331)	**0.230**	(0.139–0.321)	**0.222**	(0.135–0.310)	0.076	(−0.014 to 0.165)	**0.134**	(0.044–0.224)	**0.145**	(0.046–0.244)
Emergency department visit	Probability of visit											**−0.042**	(−0.081 to −0.003)
	Total number of visits											**−0.089**	(−0.158 to −0.021)
Outpatient visit	Probability of visit			**−0.128**	(−0.187 to −0.069)	−**0.215**	(−0.267 to −0.163)	**−0.151**	(−0.196 to −0.106)	**−0.70**	(−1.167 to −0.233)	**−0.226**	(−0.277 to −0.175)
	Total number of visits					**−0.123**	(−0.180 to −0.065)	**−0.091**	(−0.138 to −0.044)			**−0.104**	(−0.154 to −0.054)
	Conditional number of visits			**0.074**	(0.009–0.139)								

*Notes*: CI is the Concentration index, HI is the horizontal inequity index, and Con.I denotes confidence interval; 95% confidence interval in parentheses, bold figures denote significance at 5% level of significance; HIs are only reported if CIs are statistically significant at 95% level of significance.

### Inequity in inpatient admission

All HI indices reported in Table 2 for any type of hospital admission were positive and significant, suggesting a pro-rich inequity in the utilization of these services. In other words, people of more advantaged SES used more in-patient hospital services after accounting for differences in need.

However, the results in Table 3 distinguish that private hospital admissions were the driver of inequity in hospital admissions. CIs for public hospital admissions were not statistically significant, demonstrating that there was no inequity in public in-patient services due to eating disorders. In contrast, CIs and HI indices for most measures of private hospital service utilization were positive and significant. HI indices for the probability of private hospital admission ranged from 0.15 to 0.28, with a decreasing trend in these HI indices from 2005 to 2020. This suggests that individuals of more advantaged SES were more likely to have private inpatient admissions, and they visited more frequently.

**Table 3.** Inequality and inequity indices of public and private hospital admissions due to eating disorders

		Inequality (CI, 95% confidence interval)											
		2005		2008		2011		2014		2017		2020	
Type of healthcare utilization	Type of visit	CI	95%Con.I	CI	95%Con.I	CI	95%Con.I	CI	95%Con.I	CI	95%Con.I	CI	95%Con.I
Public hospital admission	Probability of visit	**−0.088**	(−0.141 to −0.036)	−0.012	(−0.061 to 0.038)	−0.012	(−0.055 to 0.030)	0.034	(−0.002 to 0.071)	−0.004	(−0.045 to 0.036)	−0.011	(−0.055 to 0.034)
	Total number of visits	0.101	(−0.062 to 0.264)	−0.015	(−0.098 to 0.068)	−0.003	(−0.079 to 0.072)	0.021	(−0.168 to 0.211)	0.017	(−0.051 to 0.084)	−0.037	(−0.109 to 0.034)
	Conditional number of visits	**0.201**	(0.052–0.351)	−0.001	(−0.057 to 0.054)	0.014	(−0.034 to 0.062)	−0.027	(−0.209 to 0.154)	0.021	(−0.023 to 0.066)	−0.023	(−0.071 to 0.025)
Private hospital admission	Probability of visit	**0.209**	(0.166–0.252)	**0.289**	(0.244–0.334)	**0.208**	(0.166–0.250)	**0.166**	(0.133–0.199)	**0.144**	(0.110–0.178)	**0.177**	(0.142–0.212)
	Total number of visits	**0.403**	(0.241–0.566)	**0.493**	(0.375–0.611)	**0.399**	(0.290–0.507)	**0.317**	(0.205–0.428)	**0.317**	(0.192–0.443)	**0.388**	(0.242–0.534)
	Conditional number of visits	0.048	(−0.093 to 0.188)	**0.101**	(0.003–0.199)	**0.126**	(0.033–0.220)	0.002	(−0.093 to 0.096)	0.065	(−0.043 to 0.173)	0.003	(−0.123 to 0.128)
		Inequity (HI, 95% confidence interval)											
		HI	95%Con.I	HI	95%Con.I	HI	95%Con.I	HI	95%Con.I	HI	95%Con.I	HI	95%Con.I
Public hospital admission	Probability of visit	**−0.091**	(−0.146 to −0.037)										
	Conditional number of visits	**0.176**	(0.030–0.323)										
Private hospital admission	Probability of visit	**0.210**	(0.093–0.327)	**0.277**	(0.093–0.327)	**0.205**	(0.093–0.327)	**0.150**	(0.093–0.327)	**0.157**	(0.093–0.327)	**0.161**	(0.093–0.327)
	Total number of visits	**0.403**	(0.242–0.565)	**0.493**	(0.376–0.609)	**0.399**	(0.292–0.505)	**0.317**	(0.206–0.427)	**0.317**	(0.194–0.441)	**0.388**	(0.244–0.533)
	Conditional number of visits			**0.098**	(0.003–0.193)	**0.150**	(0.060–0.240)						

*Notes*: CI is the Concentration index, HI is the horizontal inequity index, and Con.I denotes confidence interval; 95% confidence interval in parentheses, bold figures denote significance at 5% level of significance; HIs are only reported if CIs are statistically significant at 95% level of significance.

### Decomposition analysis

**Figure 1.**
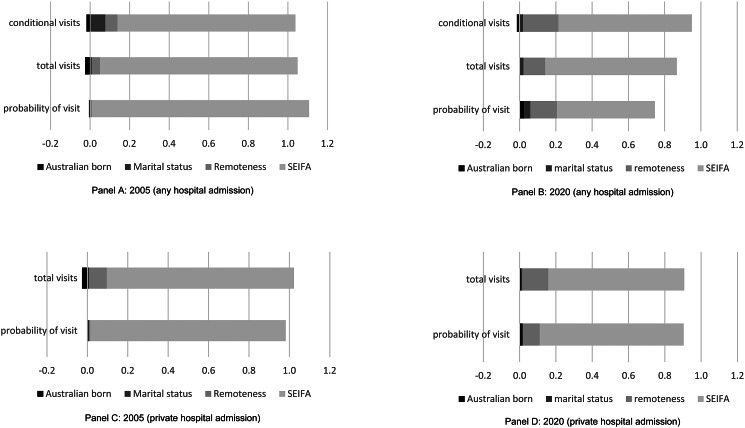
Contributing factors of inequity in any hospital (public/private) and private hospital admission among people with eating disorders in New South Wales.

The decomposition analyses in Table 4 and online Supplementary Table S6 (and Fig. 1) demonstrate that SES and the location of residence were the largest drivers of the pro-rich inequity in any hospital (public or private) and private hospital admissions between 2005 and 2020, respectively. Although SES remained the largest contributor to inequity over the study period, examination of trends shows the contribution of area of residence to the inequity in private hospital admissions increased from 3% to 9% between 2005 and 2020, while the contribution of SES decreased (Table 4). Decomposition analyses for emergency department and outpatient visits also found that SES was the main contributor to the inequities (online Supplementary Tables S7 and S8).

**Table 4.** Decomposition analysis of eating disorder-related private hospital inpatient care

	2005				2020			
	Probability of visit		Total number of visits		Probability of visit		Total number of visits	
	CCI	% contr.	CCI	% contr.	CCI	% contr.	CCI	% contr.
**Need variables**								
Female	−0.002	−1.0%	−0.005	−1.3%	0.001	0.8%	0.004	1.0%
Age (Ref: less than 15 years)								
15–24 years	0.000	−0.2%	−0.001	−0.2%	0.002	1.1%	0.003	0.8%
25–34 years	0.001	0.6%	0.004	0.9%	0.002	1.3%	0.005	1.4%
35–44 years	0.000	0.0%	0.000	0.1%	0.000	0.0%	0.000	−0.1%
45 years and above	0.000	0.0%	0.000	0.1%	−0.001	−0.7%	−0.003	−0.9%
*Subtotal of age*	*0*.*001*	*0.4%*	*0*.*003*	*0.9%*	*0*.*003*	*1.7%*	*0*.*005*	*1.2%*
Comorbidities (Ref: no common psychiatric or medical comorbidity)								
One or more common psychiatric comorbidity	0.000	0.0%	0.002	0.6%	0.001	0.4%	0.000	−0.1%
One or more common medical comorbidity	0.000	−0.1%	−0.001	−0.1%	0.000	0.0%	0.001	0.1%
One or more common psychiatric and medical comorbidity	0.002	0.8%	0.002	0.6%	0.006	3.3%	0.006	1.6%
*Subtotal of comorbidity*	*0*.*002*	*0.7%*	*0*.*003*	*1.1%*	*0*.*007*	*3.7%*	*0*.*007*	*1.6%*
Duration of any eating disorder (ref: less than 1 year)								
1–3 years	−0.002	−1.0%	−0.004	−1.0%	0.000	0.2%	0.002	0.5%
Over 3 years	0.000	0.1%	0.002	0.6%	0.005	3.1%	0.019	4.9%
*Subtotal of duration of any eating disorder*	*−0*.*002*	*−0.9%*	*−0*.*002*	*−0.4%*	*0*.*005*	*3.3%*	*0*.*021*	*5.4%*
**Total contribution of need variables**	**−0**.**001**	**−0.8%**	**−0**.**001**	**0.3%**	**0**.**016**	**9.5%**	**0**.**037**	**9.2%**
**Non-need variables**								
Australia born	0.000	0.0%	−0.010	−2.6%	0.002	1.1%	0.003	0.7%
Marital status (ref: never married)								
Married or de-facto	0.000	0.2%	0.001	0.2%	0.000	0.0%	0.000	0.1%
Widowed/divorced/separated	0.001	0.6%	0.003	0.7%	0.002	0.8%	0.003	0.6%
*Subtotal of marital status*	*0*.*001*	*0.8%*	*0*.*004*	*0.9%*	*0*.*002*	*0.8%*	*0*.*003*	*0.7%*
Remoteness (Ref: major city)								
Regional or remote	*0*.*006*	*3.1%*	*0*.*035*	*8.7%*	*0*.*016*	*9.2%*	*0*.*056*	*14.4%*
Socioeconomic status (SEIFA) (Ref: Decile 1: Most disadvantaged)								
Decile 2	0.004	2.1%	0.020	4.8%	−0.004	−2.1%	0.011	2.8%
Decile 3	0.001	0.6%	0.002	0.6%	−0.002	−1.2%	0.010	2.5%
Decile 4	−0.001	−0.6%	0.001	0.2%	−0.006	−3.4%	0.011	2.8%
Decile 5	−0.006	−3.1%	0.002	0.6%	−0.003	−1.5%	−0.004	−1.0%
Decile 6	0.000	−0.1%	0.008	1.9%	−0.001	−0.5%	−0.002	−0.5%
Decile 7	0.000	0.0%	0.000	−0.1%	0.002	0.9%	−0.004	−1.0%
Decile 8	0.018	8.5%	0.019	4.7%	0.010	5.4%	0.022	5.7%
Decile 9	0.047	22.3%	0.065	16.2%	0.059	33.3%	0.126	32.4%
Decile 10 (Least disadvantaged)	0.140	67.1%	0.257	63.8%	0.086	48.4%	0.121	31.2%
*Subtotal of socioeconomic status*	*0*.*203*	*96.8%*	*0*.*374*	*92.7%*	*0*.*141*	*79.3%*	*0*.*291*	*74.9%*
**Total contribution of non-need variables**	**0**.**210**	**100.7%**	**0**.**403**	**99.7%**	**0**.**161**	**90.4%**	**0**.**353**	**90.7%**
Residual	0.000	0.0%	0.001	0.2%	0.000	0.0%	−0.002	−0.5%

*Notes*: CCI, Contribution to the concentration index; contr., contribution; CIs for conditional number of private inpatient admissions in 2005 and 2020 were not significant, and hence, no decomposition analyses were shown; residuals refer to unexplained components after accounting for the effects of the explanatory variables included in the model; contribution of need variables, non-need variables and residual factor sum to 100%. Please note that percentage may not total to 100% due to rounding in some cases.

## Discussion

This study investigated the socioeconomic inequity in healthcare utilization for eating disorders in NSW. Results for all hospital admissions indicate a pro-rich inequity; however, this result is driven by socioeconomic inequity in private hospital admissions for eating disorders, and there was no significant inequity in public hospital admissions. Results further indicate public hospital emergency department visits were also equitable across the period, except for 2020, when a pro-poor inequity was observed. Visits to public outpatient services were concentrated among individuals of more disadvantaged SES for the majority of the study period (2008 to 2020). Further, inequities in eating disorder-related private hospital admissions, emergency department visits and public outpatient visits were mainly explained by SES and remoteness of area of residence. However, while SES had the largest contribution to the inequity in private hospital admissions, the contribution of remoteness of residence more than doubled over the study period.

The pro-rich inequity in all hospital admissions for people with eating disorders observed in the current study is not consistent with previous research, which showed no significant inequity in Australian general population inpatient admissions for any medical or psychiatric condition in 2011–12 and 2014–15 (Pulok et al., 2020a). This suggests that socioeconomic inequity in inpatient admissions for an eating disorder is higher than that for inpatient admissions due to any reason in Australia, and this is consistent with a similar study in the United Kingdom (Mangalore, Knapp, & Jenkins, 2007). However, further analysis reveals that the source of this inequity was limited to private hospital admissions – there was no evidence of inequities in public hospital admissions. That SES is the largest driver of pro-rich inequity in private hospital admissions is perhaps an unsurprising finding, and is consistent with Australian data displaying pro-rich inequity in accessing specialist services (Pulok et al., 2020b). However, horizontal inequity in private hospital admissions is a critical issue in eating disorders because a large proportion of higher-level care is fulfilled by the private hospital system (Australian Institute of Health and Welfare, 2024; Butterfly Foundation, 2024). Although specialist tertiary eating disorders care is available in public hospitals, bed availability is limited (Maguire & Maloney, 2021), and in-patient admissions for eating disorder care are usually reserved for the most acute cases. This means that public hospital admissions for eating disorders can be difficult to access, and dedicated private facilities for eating disorders may be easier to access if individuals have private health insurance. The current results indicate a significant pro-rich distribution in terms of the frequency of private inpatient admissions after adjusting for need.

Compared with existing mental healthcare research, our finding of pro-rich inequity in private inpatient admissions is in line with other Australian data that demonstrates pro-rich inequity in psychiatric care utilization from 2009 to 2017 (Hashmi et al., 2023) and for inpatient admissions due to any psychiatric cause in the Household, Income and Labour Dynamics in Australia survey (Goodall & Scott, 2008). Similar to our findings, pro-rich inequity was also found for hospital admissions when the patients were admitted as private patients in public hospitals – a unique option available in the Australian healthcare system for those who have private health insurance (Van Doorslaer, Clarke, Savage, & Hall, 2008). Rates of private health insurance are higher in those with higher SES, and this is a parsimonious explanation for the current data.

The current results show that inequity in private hospital admissions is not a recent development and has remained fairly stable over the 15-year study period. However, results of the decomposition analysis show a key change over the period – remoteness of residence accounted for a relatively larger portion of socioeconomic inequity in private hospital admissions over time. Although SES remained the largest contributor, the contribution of location of residence to the pro-rich inequities in the probability and total private hospital admissions increased between 2005 and 2020. The effect of remoteness could be indirectly related to SES, as residents of major cities are, on average, more likely to have higher income and, therefore, more ability to pay for private health insurance and the gap fees or co-payments incurred at private hospitals. However, the current data controls for SES in the decomposition analysis of remoteness of residence, and, therefore, cannot be dismissed as simply the product of individuals living in major cities having higher SES. After accounting for differences in SES, the current data show remoteness of residence is an increasing barrier to accessing private hospital care for eating disorders. These results further add to the evidence that barriers to accessing eating disorder services are significantly exacerbated in regional and remote areas of Australia (Butterfly Foundation, 2020).

Overall, the results for the public healthcare sector are positive, and indicate broad equity in services for eating disorders in NSW. There was no evidence of inequities in public hospital admissions and, broadly, the same for emergency department visits for people with eating disorders. The current results demonstrate public outpatient services were more frequently used by those with relatively lower SES. Outpatient services are critical to eating disorders care, because most of the available evidence-based treatments for eating disorders are long-term, community-based treatments (Hay et al., 2014). Ideally, timely access to good community care can provide the necessary support to help people with eating disorders in their recovery and avoid the need for emergency department visits or in-patient admissions. But, for those who need higher-level care, being engaged with community outpatient services can provide a pathway to step-up to more intensive hospital services. Outpatient services are also critical in step-down care after hospital treatment, as further community care is needed to support psychological recovery, maintain the medical stability provided by in-patient services, and support long-term functional recovery (Pehlivan et al., 2022).

As the current study includes only public outpatient services, a plausible explanation for the results is that individuals with relatively higher SES were more likely to utilize private community outpatient services than public services. Private psychologists provide a large proportion of the long-term community care for people with eating disorders – they can offer more flexibility and can be easier to access than public outpatient services, so for individuals who can afford the co-payment, they may be preferable. This could result in public outpatient care for eating disorders being concentrated among those from relatively lower SES, and is consistent with the principle that financial contributions made by patients should be according to their ability to pay (Fleurbaey & Schokkaert, 2011; Wagstaff & van Doorslaer, 2000), and the aims of distributional efficacy in public healthcare. However, in light of the data as a whole, a potential ‘hidden’ inequality in the pro-poor inequity observed in use of public outpatient services, could be that public outpatient services are holding the care for people with eating disorders with relatively lower SES for longer, and perhaps over a wider spectrum of severity, due to limited availability of public inpatient hospital care, and the financial or regional inaccessibility of alternative private services. If so, the increasing inequity in private inpatient admissions for individuals with eating disorders in Australia may be contributing to the overall economic burden of eating disorders.

### Strengths and limitations

The current study is the first to provide data on the extent of socioeconomic inequity in private hospital admissions for eating disorders in NSW. There is limited information about the extent of socioeconomic inequity in healthcare utilization in Australia, and no published studies investigating socioeconomic inequality and inequity for different types of healthcare for eating disorders. The lack of evidence could be due to less attention on eating disorder-specific research compared to other mental health areas in Australia (Bryant et al., 2023). A key strength is the use of administrative health data, a stable and reliable source that overcomes the limitations of survey data, such as recall bias, lack of specific detail, and measurement errors. In addition, we were able to adjust for need factors, which are often unavailable in survey data. Horizontal equity in healthcare service provision is an important policy concern for many Organisation for Economic Co-operation and Development (OECD) countries, particularly for countries such as Australia with a tax-financed universal health insurance system (Leeder, 2003). Employing the CI approach to administrative health data is also relatively new in mental health research, although the importance of administrative data in developing equity indicators for healthcare services has received increasing attention in Australia and Canada (Doiron, Raina, Fortier, Linkage Between, & Health Care Utilization Data: Meeting of Canadian Stakeholders workshop, 2013; Olver, 2014). Another strength is that the study differentiated between high and low users in terms of frequency of hospital admissions, emergency department or outpatient visits, while measuring the extent of the inequity. The extent of inequity can be different between probability and intensity of use (Doorslaer, Koolman, & Jones, 2004), which was demonstrated separately in our analysis. Separate analysis of public and private hospital admissions is another novel contribution, and the inclusion of trend analysis in socioeconomic inequity was a significant strength.

A limitation of administrative data is that there is limited information on individual-level SES. SEIFA incorporates the average income of the population within a certain geography, which may not reflect individual-level SES. However, this index is extensively used in many studies examining the association between SES and different social outcomes in Australia (Johar, Jones, & Savage, 2014). Although NSW administrative health data is a high-quality source, hospital coding, especially in the emergency department, is limited, and the current demographic data does not include race and ethnicity. The inequity in the quality of healthcare utilization could not be considered and is a limitation of this type of research generally, as most studies have relied on standard health administrative data. The current data is limited to hospital admissions, emergency visits, and public outpatient visits by people with eating disorders and future inequalities research that includes primary health, allied health and specialist care providers is warranted. It is also important to note that people with lower SES backgrounds have been shown to be less likely to seek medical advice for eating disorder care (Forrest, Smith, & Swanson, 2017; Sonneville & Lipson, 2018), and as a consequence, are less likely to use eating disorder-related healthcare. Therefore, measures of health utilization may underestimate the overall health inequalities related to eating disorders in our communities. Future research may also explore the inequity of healthcare utilization by different types of eating disorders so that differences between groups can be better understood in policymaking. In addition, we could not investigate the extent of the socioeconomic inequity in private outpatient visits due to the lack of availability and access to such data in NSW. Whether long-term outpatient-based intervention yields more favorable outcomes than hospital admissions is beyond the scope of the current study.

## Conclusions

Ensuring health equity – by identifying, quantifying and addressing systematic unfairness – is the first step to health equality. The current research indicates broad equity in public healthcare services for eating disorders in NSW but observes significant pro-rich inequity in private hospital admissions. Horizontal inequity in private inpatient care is an important systemic issue for the eating disorders sector, as a large proportion of hospital care is undertaken in private facilities. Although SES had the largest contribution to the inequity in private hospital admissions, decomposition analysis shows inequity due to remoteness of residence increased over the study period. The current data, therefore, adds to existing evidence of greater barriers to care for people with eating disorders living in rural and remote areas. This research evaluates the fairness of eating disorder related-healthcare in NSW and provides important information for the development of programs and policies to improve access to care for individuals with eating disorders.

## Supporting information

Ahmed et al. supplementary material 1 Ahmed et al. supplementary material

Ahmed et al. supplementary material 2 Ahmed et al. supplementary material

## Supplementary material

The supplementary material for this article can be found at https://doi.org/10.1017/S0033291724002290.
