# Supplementary material for: Socioeconomic inequity in the utilization of healthcare among people with eating disorders in Australia
Source: Psychol Med. 2024 Oct 4;54(14):3863–75. doi: 10.1017/S0033291724002290 (PMC11578912; doi:10.1017/S0033291724002290)
Supplement: Ahmed et al. supplementary material 1 — Ahmed et al. supplementary material [file S0033291724002290sup001.docx]

# Appendix

## Socioeconomic Advantage and Disadvantage (SEIFA):

Socioeconomic Advantage and Disadvantage (SEIFA) ranks areas in Australia based on their relative socioeconomic advantage and disadvantage, considering factors such as income, education, occupation, employment, and housing characteristics. There are four indices of SEIFA, including the Index of Relative Socioeconomic Advantage and Disadvantage (IRSAD), which summarises the economic and social conditions of people and households within an area, including both advantage and disadvantage measures (Australian Bureau of Statistics, 2008; 2013). The IRSAD (Australian Bureau of Statistics, 2008; 2013) was employed in this study, consistent with recent studies (Islam, Ormsby, Kabir, & Khanam, 2021; Pulok, van Gool, & Hall, 2020a; 2020b). SEIFA scores are categorised by decile, with the lowest decile (1) representing the most disadvantaged and the highest (10) representing the least disadvantaged.

## Estimation of Concentration Index (CI) and Horizontal Inequity (HI) index:

Concentration Indices (CIs) in this study are calculated using the following “convenient regression” approach suggested by Kakwani et al. (1997) (Kakwani, Wagstaff, & van Doorslaer, 1997):

$$2\sigma_{r}^{2}(\frac{h_{i}}{\mu})=\alpha+\beta r_{i}+\varepsilon_{i}$$

where $h_{i}$ is the healthcare resource utilisation variable, $r_{i}$is the fractional ranking of individuals by SES, which is SEIFA IRSAD in the present study, $\mu$ is the population mean of healthcare resource utilisation (cost), $\varepsilon_{i}$was the random error term and $\sigma_{r}^{2}$is the population variance of fractional rank. The ordinary least squares (OLS) estimate of $\beta$ represents the CI.

Following this, CIs need to be normalised to allow for comparing inequalities across different populations and time periods, and the correction is particularly important for binary outcomes (Wagstaff, 2005). To address this issue, two approaches can be taken: Wagstaff's approach, which normalises the CI by dividing it by one minus the mean of the healthcare use variable or Erreygers's correction, which corrects the CI by multiplying it by four times the mean healthcare use (Erreygers, 2009). In this study, Erreygers's correction was employed, which satisfied all four properties of rank-dependent measures of inequality (Kjellsson & Gerdtham, 2013).

# References

Australian Bureau of Statistics (2008). *Information Paper: An Introduction to Socio-Economic Indexes for Areas (SEIFA),2006*. Retrieved from <https://www.abs.gov.au/ausstats/abs@.nsf/Latestproducts/2039.0Main%20Features62006?opendocument&tabname=Summary&prodno=2039.0&issue=2006&num=&view=#Figure%204.1%20IRSD%20Scores%20Histogram>

Australian Bureau of Statistics (2013). *Socio-Economic Indexes for Areas (SEIFA) - Technical Paper*. Retrieved from <https://www.abs.gov.au/ausstats/abs@.nsf/DetailsPage/2033.0.55.0012011?OpenDocument>

Erreygers G. (2009). Correcting the Concentration Index. *Journal of Health Economics*, 28(2)**,**  504-515. doi: 10.1016/j.jhealeco.2008.02.003.

Islam M.I., Ormsby G.M., Kabir E., & Khanam R. (2021). Estimating income-related and area-based inequalities in mental health among nationally representative adolescents in Australia: The concentration index approach. *PloS One*, 16(9)**,**  e0257573. doi: 10.1371/journal.pone.0257573.

Kakwani N., Wagstaff A., & van Doorslaer E. (1997). Socioeconomic inequalities in health: Measurement, computation, and statistical inference. *Journal of Econometrics*, 77(1)**,**  87-103. doi: 10.1016/S0304-4076(96)01807-6.

Kjellsson G., & Gerdtham U.-G. (2013). On correcting the concentration index for binary variables. *Journal of Health Economics*, 32(3)**,**  659-670. doi: 10.1016/j.jhealeco.2012.10.012.

Pulok M.H., van Gool K., & Hall J. (2020a). Horizontal inequity in the utilisation of healthcare services in Australia. *Health Policy*, 124(11)**,**  1263-1271. doi: 10.1016/j.healthpol.2020.08.012.

Pulok M.H., van Gool K., & Hall J. (2020b). Inequity in physician visits: the case of the unregulated fee market in Australia. *Social Science and Medicine*, 255**,**  113004. doi: 10.1016/j.socscimed.2020.113004.

Wagstaff A. (2005). The bounds of the concentration index when the variable of interest is binary, with an application to immunization inequality. *Health Economics*, 14(4)**,**  429-432. doi: 10.1002/hec.953.
