# Supplementary material for: Socioeconomic inequity in the utilization of healthcare among people with eating disorders in Australia
Source: Psychol Med. 2024 Oct 4;54(14):3863–75. doi: 10.1017/S0033291724002290 (PMC11578912; doi:10.1017/S0033291724002290)
Supplement: Ahmed et al. supplementary material 2 — Ahmed et al. supplementary material [file S0033291724002290sup002.docx]

# Supplementary Tables

## Supplementary Table 1: Eating disorder-related diagnostic codes used to identify patients with eating disorders during hospital admissions or emergency department/ ambulatory visits

| **Type of eating disorder** | **Codes** | **Type of code** | **Description** |
| --- | --- | --- | --- |
| **Anorexia Nervosa** | F50.0 | ICD-10 AM | Anorexia nervosa |
|  | F50.1 | ICD-10 AM | Atypical anorexia nervosa |
|  | 307.1 | ICD-9 CM | Anorexia nervosa |
|  | 56882008 | SNOMED CT | Anorexia nervosa |
|  | 63393005 | SNOMED CT | Anorexia nervosa, binge-eating purging type |
|  | 77675002 | SNOMED CT | Anorexia nervosa, restricting type |
|  | 161471008 | SNOMED CT | H/O: anorexia nervosa |
|  | 231522009 | SNOMED CT | Atypical anorexia nervosa |
|  | 403313001 | SNOMED CT | Hypertrichosis in anorexia nervosa |
|  | 698695006 | SNOMED CT | Anorexia nervosa in remission^+^ |
|  | 722959000 | SNOMED CT | Anorexia nervosa co-occurrent with dangerously low body weight |
|  | 722960005 | SNOMED CT | Anorexia nervosa co-occurrent with significantly low body weight |
|  | 723917005 | SNOMED CT | Dangerously low body weight co-occurrent and due to anorexia nervosa of restricting type |
|  | 723918000 | SNOMED CT | Dangerously low body weight co-occurrent and due to anorexia nervosa of binge-eating purging type |
|  | 723919008 | SNOMED CT | Significantly low body weight co-occurrent and due to anorexia nervosa of restricting type |
| **Bulimia Nervosa** | F50.2 | ICD-10 AM | Bulimia nervosa |
|  | F50.3 | ICD-10 AM | Atypical bulimia nervosa |
|  | 307.51 | ICD-9 CM | Bulimia nervosa |
|  | 32721004 | SNOMED CT | Bulimia nervosa, purging type |
|  | 59645001 | SNOMED CT | Bulimia nervosa, non-purging type |
|  | 78004001 | SNOMED CT | Bulimia nervosa |
|  | 231523004 | SNOMED CT | Atypical bulimia nervosa |
|  | 248123000 | SNOMED CT | Pattern of overeating and vomiting (bulimia) |
|  | 698698008 | SNOMED CT | Bulimia nervosa in remission |
|  | 126151000119107 | SNOMED CT | H/O: bulimia nervosa |
|  | F50.4 | ICD-10 AM | Overeating associated with other psychological disturbances |
|  | F50.5 | ICD-10 AM | Vomiting associated with other psychological disturbances |
| **Other/ Unspecified Eating Disorders** | F50.9 | ICD-10 AM | Eating disorder unspecified |
|  | F50.8 | ICD-10 AM | Other eating disorders |
|  | 307.5 | ICD-9 CM | Other and unspecified disorders of eating |
|  | 307.54 | ICD-9 CM | Psychogenic vomiting |
|  | 248122005 | SNOMED CT | Bingeing |
|  | 270902002 | SNOMED CT | Overeating associated with other psychological disturbances |
|  | 275474009 | SNOMED CT | Psychogenic overeating |
|  | 407666007 | SNOMED CT | Nocturnal sleep-related eating disorder |
|  | 439960005 | SNOMED CT | Binge eating disorder |

Notes: All of these codes were used to identify eating disorder cases. ICD-10 AM codes were used to identify hospital usage (private and public), whereas emergency department visits due to eating disorders were determined using ICD-10 AM, ICD-9 CM or SNOMED CT codes. Outpatient visits were identified from ICD-10 AM diagnosis codes available in public outpatient data.

+ The inclusion of “Anorexia Nervosa in remission” was for the identification of people with an eating disorder (past or present) only from the emergency department data. We note that this eating disorder diagnosis “in remission” suggests the condition is no longer present and that the patterns of healthcare utilisation are likely to be different for those with a current versus past diagnosis of AN. We have run the analysis and found no individual with “Anorexia Nervosa in remission (SNOMED CT)” from the emergency data. Therefore, the inclusion of this SNOMED code does not affect our analysis.

## Supplementary Table 2: Outcome variables

| **Type of healthcare service utilisation** | **Type of utilisation** | **outcome variable** | **Description** |
| --- | --- | --- | --- |
| Public/ Private/ Any Hospital admission | Probability of admission | Dichotomous variable | Takes a value of one if the individual had an eating disorder-related hospital admission for a given year or zero otherwise |
|  | Total number of admissions | Non-negative Count variable | non-negative admissions to hospitals, including zero admissions |
|  | Conditional number of admissions | Positive count variable | Includes positive numbers of hospital admissions, conditional on having at least one admission for a given year |
| Emergency department visit | Probability of visit | Dichotomous variable | Takes a value of one if the individual had an eating disorder-related emergency department visit for a given year or zero otherwise |
|  | Total number of visits | Non-negative Count variable | non-negative emergency department visits, including zero visit |
|  | Conditional number of visits | Positive count variable | Includes positive numbers of emergency department visits, conditional on having at least one emergency department visit for a given year |
| Outpatient visit | Probability of visit | Dichotomous variable | Takes a value of one if the individual had an eating disorder-related outpatient visit for a given year or zero otherwise |
|  | Total number of visits | Non-negative Count variable | non-negative outpatient visit, including zero visit |
|  | Conditional number of visits | Positive count variable | Includes positive numbers of outpatient visits, conditional on having at least one outpatient visit for a given year |

## Supplementary Table 3: Common psychiatric and medical comorbidities associated with eating disorders and their ICD-10 AM diagnostic codes

| **Comorbidity** | **ICD-10 AM** |
| --- | --- |
| **Psychiatric comorbidity** |  |
| 1. mood disorders | F30-F39 |
| 1. anxiety disorders | F40-48 |
| 1. post-traumatic stress disorder (PTSD) and trauma | F43.1 |
| 1. substance use disorders | F10-19 |
| 1. personality disorders | F60-69 |
| 1. suicide ideation | R45.81 |
| **Medical comorbidity** |  |
| 1. type 1 and 2 diabetes | E10, E11 |
| 1. cardiovascular complications | I11-13, I20-28, I34-36, I42, I44, I46-I51, I61-I67, I69, I70-I77, I80, G45, G46 |
| 1. osteopenia and osteoporosis | M80, M81, M82 |
| 1. gastrointestinal problems   [Oesophageal disorder bowel disorder  anorectal disorders] | I98.2, I98.3  K59  K62 |
| 1. joint pains | M25.5 |
| 1. headache and migraine | R51, G43 |

## Supplementary Table 4: Details of individual need and non-need variables

| **Variable** | **Type** | **Description** |
| --- | --- | --- |
| **Need variables** |  |  |
| Sex | Dichotomous | Sex was categorised into two groups: male and female, with males as a reference group. |
| Age | Categorical | Age was divided into 5 groups: less than 15 years (reference category), 15-24 years, 25-34 years, 35-44 years, and 45 years and above. |
| Duration of eating disorder | Categorical | Duration of any eating disorder was categorised into three groups: less than 1 year (reference category), 1-3 years, and over 3 years. The duration was calculated from the first contact (APDC/ EDDC/ MHAMB) with eating disorder. |
| Psychiatric and medical comorbidities | Categorical | Psychiatric and medical comorbidities were divided into four groups: 1) had no common psychiatric or medical comorbidity (reference group); 2) had one or more common psychiatric comorbidity; 3) had one or more medical comorbidity, comorbidity; and 4) had one or more common psychiatric and medical comorbidity. |
| **Non-need variables** |  |  |
| Born in Australia | Dichotomous | Born in Australia is a dichotomous variable with a value of 1 if born in Australia and 0 otherwise |
| Marital status | Categorical | The marital status variable had three groups: never married (reference), married or in a de-facto relationship, and widowed/ divorced. |
| Remoteness of residence | Dichotomous | The remoteness of residence was categorised into Major city (reference group) and Regional or remote. |
| Socioeconomic status [SEIFA (IRSAD)] | Categorical | Socioeconomic Indexes for Areas (SEIFA) 2011 version based on NSW was used to measure socioeconomic status. Among different SEIFA indexes, the Index of Relative Socio-economic Advantage and Disadvantage (IRSAD) was used. SEIFA was categorised into ten groups, with decile 10 (least disadvantaged group) as a reference category. |

Notes: SEIFA: Socioeconomic Indexes for Areas, IRSAD: Index of Relative Socio-economic Advantage and Disadvantage, APDC: Admitted Patient Data Collection, EDDC: Emergency Department Data Collection

## Supplementary Table 5: Total frequency and proportion/mean of hospital visits for people with eating disorders

| **Types of healthcare utilisation** | **Type of visit** | **2005** | **2008** | **2011** | **2014** | **2017** | **2020** |
| --- | --- | --- | --- | --- | --- | --- | --- |
| Hospital admission | Probability of visit | 1291 (0.321,0.467) | 1374 (0.353,0.478) | 1715 (0.354,0.478) | 2222 (0.298,0.458) | 2051 (0.311,0.463) | 1744 (0.319,0.466) |
|  | Total number of visits | 1291 (1.805,7.125) | 1374 (1.999,6.867) | 1715 (3.043,11.089) | 2222 (1.863,7.699) | 2051 (1.580,6.257) | 1744 (1.176,4.661) |
|  | Conditional number of visits | 414 (5.628,11.705) | 485 (5.662,10.630) | 607 (8.598,17.319) | 663 (6.243,13.096) | 638 (5.078,10.402) | 556 (3.689,7.677) |
| Emergency department visit | Probability of visit | 1291 (0.060,0.237) | 1374 (0.057,0.231) | 1715 (0.102,0.303) | 2222 (0.108,0.311) | 2051 (0.145,0.352) | 1744 (0.154,0.361) |
|  | Total number of visits | 1291 (0.068,0.310) | 1374 (0.071,0.395) | 1715 (0.120,0.381) | 2222 (0.140,0.460) | 2051 (0.174,0.470) | 1744 (0.181,0.473) |
|  | Conditional number of visits | 77 (1.143,0.622) | 78 (1.244,1.142) | 175 (1.171,0.435) | 241 (1.286,0.693) | 298 (1.195,0.547) | 269 (1.175,0.535) |
| Outpatient visit | Probability of visit | 1291 (0.485,0.500) | 1374 (0.489,0.500) | 1715 (0.513,0.500) | 2222 (0.590,0.492) | 2051 (0.538,0.499) | 1744 (0.571,0.495) |
|  | Total number of visits | 1291 (5.858,13.920) | 1374 (7.631,18.152) | 1715 (7.546,16.329) | 2222 (10.333,20.564) | 2051 (9.433,18.852) | 1744 (12.650,23.796) |
|  | Conditional number of visits | 626 (12.081,18.018) | 672 (15.603,23.444) | 879 (14.722,20.365) | 1310 (17.527,24.317) | 1104 (17.524,22.773) | 995 (22.173,27.956) |

Note: mean and standard deviations are in parentheses

## Supplementary Table 6: Decomposition analysis of eating disorder-related any hospital inpatient care (public or private) in 2005 and 2020

|  | **2005** | | | | | | **2020** | | | | | |
| --- | --- | --- | --- | --- | --- | --- | --- | --- | --- | --- | --- | --- |
|  | **Probability of visit** | | **Total number of visits** | | **Cond. Number of visits** | | **Probability of visit** | | **Total number of visits** | | **Cond. Number of visits** | |
|  | CCI | % contr. | CCI | % contr. | CCI | % contr. | CCI | % contr. | CCI | % contr. | CCI | % contr. |
| **Need variables** |  |  |  |  |  |  |  |  |  |  |  |  |
| Female | -0.002 | -1.6% | -0.005 | -1.5% | 0.009 | 3.8% | 0.002 | 1.7% | 0.002 | 1.0% | 0.000 | 0.2% |
| Age (Ref: less than 15 years) |  |  |  |  |  |  |  |  |  |  |  |  |
| 15-24 years | 0.000 | 0.4% | 0.000 | 0.0% | 0.002 | 0.7% | -0.004 | -3.3% | 0.001 | 0.6% | 0.007 | 4.5% |
| 25-34 years | -0.004 | -3.6% | -0.001 | -0.4% | 0.001 | 0.5% | -0.006 | -4.3% | 0.000 | 0.2% | 0.000 | -0.3% |
| 35-44 years | 0.000 | -0.3% | 0.000 | 0.0% | -0.004 | -1.7% | 0.006 | 4.3% | -0.001 | -0.3% | -0.003 | -2.3% |
| 45 years and above | -0.003 | -2.7% | -0.002 | -0.5% | -0.001 | -0.6% | 0.011 | 7.8% | 0.001 | 0.4% | -0.003 | -1.8% |
| *Subtotal of age* | *-0.007* | *-6.2%* | *-0.003* | *-0.9%* | *-0.002* | *-1.1%* | *0.007* | *4.5%* | *0.001* | *0.9%* | 0.000 | 0.3% |
| Comorbidities (Ref: No common psychiatric or medical comorbidity) |  |  |  |  |  |  |  |  |  |  |  |  |
| One or more common psychiatric comorbidity | -0.003 | -3.2% | 0.000 | -0.1% | 0.001 | 0.3% | 0.000 | 0.1% | 0.000 | 0.0% | 0.000 | 0.3% |
| One or more common medical comorbidity | -0.001 | -1.1% | -0.001 | -0.3% | -0.002 | -0.7% | 0.003 | 2.2% | 0.001 | 0.3% | 0.000 | -0.1% |
| One or more common psychiatric and medical comorbidity | 0.002 | 1.5% | 0.003 | 0.9% | 0.002 | 0.9% | 0.018 | 13.4% | 0.017 | 6.6% | 0.001 | 0.3% |
| *Subtotal of comorbidity* | *-0.002* | *-2.8%* | *0.002* | *0.5%* | *0.001* | *0.5%* | *0.021* | *15.7%* | *0.018* | *6.9%* | *0.001* | *0.5%* |
| Duration of any ED (ref: less than 1 year) |  |  |  |  |  |  |  |  |  |  |  |  |
| 1-3 years | -0.006 | -5.2% | -0.003 | -0.8% | 0.001 | 0.4% | 0.002 | 1.8% | -0.002 | -0.8% | 0.003 | 2.1% |
| Over 3 years | -0.001 | -1.0% | 0.001 | 0.5% | -0.001 | -0.5% | 0.003 | 1.9% | 0.013 | 5.3% | 0.005 | 3.6% |
| *Subtotal of duration of any ED* | *-0.007* | *-6.2%* | *-0.002* | *-0.3%* | *0.000* | *-0.1%* | *0.005* | *3.7%* | *0.011* | *4.5%* | *0.008* | *5.7%* |
| **Total contribution of need variables** | **-0.018** | **-16.8%** | **-0.008** | **-2.2%** | **0.008** | **3.1%** | **0.035** | **25.6%** | **0.032** | **13.3%** | **0.009** | **6.7%** |
| **Non-need variables** |  |  |  |  |  |  |  |  |  |  |  |  |
| Australia born | -0.001 | -0.5% | -0.008 | -2.5% | -0.004 | -1.8% | 0.003 | 2.4% | 0.000 | -0.1% | -0.002 | -1.5% |
| Marital status (ref: never married) |  |  |  |  | 0.003 | 1.3% |  |  |  |  |  |  |
| married or de-facto | -0.001 | -0.6% | 0.001 | 0.2% | 0.003 | 1.5% | 0.001 | 0.9% | 0.001 | 0.4% | 0.000 | 0.1% |
| widowed/divorced/separated | 0.001 | 0.5% | 0.002 | 0.6% | 0.014 | 6.2% | 0.003 | 2.6% | 0.004 | 1.7% | 0.003 | 1.8% |
| *Subtotal of marital status* | *0.000* | *-0.1%* | *0.003* | *0.8%* | *0.017* | *7.7%* | *0.004* | *3.5%* | *0.005* | *2.1%* | *0.003* | *1.9%* |
| Remoteness (Ref: major city) |  |  |  |  |  |  |  |  |  |  |  |  |
| Regional or remote | *0.008* | *7.4%* | *0.014* | *4.3%* | *0.014* | *6.2%* | *0.020* | *14.7%* | *0.030* | *12.0%* | *0.028* | *19.5%* |
| Socioeconomic status  (SEIFA) (Ref: Decile 1: Most disadvantaged) |  |  |  |  |  |  |  |  |  |  |  |  |
| Decile 2 | 0.022 | 20.4% | 0.021 | 6.6% | 0.001 | 0.5% | -0.019 | -13.8% | 0.003 | 1.2% | 0.007 | 5.0% |
| Decile 3 | 0.000 | -0.1% | -0.010 | -3.1% | -0.014 | -6.4% | 0.000 | 0.3% | 0.005 | 1.9% | 0.007 | 4.7% |
| Decile 4 | 0.009 | 8.3% | 0.004 | 1.3% | -0.004 | -1.9% | -0.014 | -10.5% | 0.001 | 0.3% | 0.010 | 7.0% |
| Decile 5 | 0.002 | 1.8% | 0.002 | 0.8% | -0.001 | -0.3% | -0.010 | -7.1% | -0.015 | -6.0% | -0.012 | -7.9% |
| Decile 6 | 0.005 | 4.3% | 0.007 | 2.3% | 0.004 | 1.6% | 0.000 | -0.1% | 0.000 | 0.0% | 0.000 | 0.0% |
| Decile 7 | -0.001 | -0.9% | 0.001 | 0.2% | -0.003 | -1.4% | 0.003 | 2.1% | 0.005 | 2.1% | 0.001 | 0.6% |
| Decile 8 | -0.003 | -3.2% | 0.013 | 4.2% | 0.006 | 2.7% | 0.004 | 2.9% | 0.005 | 1.8% | 0.002 | 1.0% |
| Decile 9 | 0.015 | 14.3% | 0.061 | 19.3% | 0.040 | 17.6% | 0.043 | 31.8% | 0.092 | 36.6% | 0.050 | 34.2% |
| Decile 10 (Least disadvantaged) | 0.070 | 65.0% | 0.215 | 68.2% | 0.175 | 77.5% | 0.065 | 48.4% | 0.087 | 34.7% | 0.042 | 29.0% |
| *Subtotal of socioeconomic status* | *0.119* | *109.9%* | *0.314* | *99.8%* | *0.204* | *89.9%* | *0.072* | *54.0%* | *0.183* | *72.6%* | *0.107* | *73.6%* |
| **Total contribution of non-need variables** | **0.126** | **116.7%** | **0.323** | **102.4%** | **0.231** | **102.0%** | **0.099** | **74.6%** | **0.218** | **86.6%** | **0.136** | **93.5%** |
| Residual | 0.000 | 0.0% | 0.000 | 0.0% | -0.013 | -5.8% | 0.001 | 0.7% | 0.001 | 0.4% | 0.001 | 0.7% |

Notes: CCI =Contribution to the concentration index, contr.= contribution, cond.=conditional; residuals refer to unexplained components after accounting for the effects of the explanatory variables included in the model; contribution of need variables, non-need variables and residual factor sum to 100%. Please note that percentage may not total to 100% due to rounding in some cases.

## Supplementary Table 7: Decomposition analysis of eating disorder-related outpatient visits in 2008 and 2020

|  | **2008** | | | | **2020** | | | |
| --- | --- | --- | --- | --- | --- | --- | --- | --- |
|  | **Probability of visit** | | **Conditional number of visits** | | **Probability of visit** | | **Total number of visits** | |
|  | CCI | % contr. | CCI | % contr. | CCI | % contr. | CCI | % contr. |
| **Need variables** |  |  |  |  |  |  |  |  |
| Female | -0.006 | 4.9% | 0.004 | 6.3% | -0.001 | 0.4% | 0.000 | 0.2% |
| Age (Ref: less than 15 years) |  |  |  |  |  |  |  |  |
| 15-24 years | -0.013 | 9.5% | 0.000 | 0.4% | -0.003 | 1.2% | 0.000 | -0.1% |
| 25-34 years | -0.012 | 8.7% | -0.001 | -0.8% | -0.005 | 2.1% | -0.001 | 0.9% |
| 35-44 years | 0.015 | -11.4% | 0.000 | -0.1% | 0.000 | -0.2% | 0.000 | -0.2% |
| 45 years and above | 0.019 | -14.5% | 0.001 | 1.7% | 0.006 | -2.4% | 0.002 | -1.8% |
| *Subtotal of age* | *0.009* | *-7.7%* | *0.000* | *1.2%* | *-0.002* | *0.7%* | *0.001* | *-1.2%* |
| Comorbidities (Ref: No common psychiatric or medical comorbidity) |  |  |  |  |  |  |  |  |
| One or more common psychiatric comorbidity | 0.000 | 0.1% | -0.015 | -21.6% | -0.005 | 2.1% | 0.000 | 0.4% |
| One or more common medical comorbidity | 0.003 | -2.5% | -0.002 | -2.3% | 0.001 | -0.3% | 0.000 | 0.0% |
| One or more common psychiatric and medical comorbidity | 0.006 | -4.4% | -0.001 | -1.5% | -0.019 | 7.9% | 0.002 | -1.7% |
| *Subtotal of comorbidity* | *0.009* | *-6.8%* | *-0.018* | *-25.4%* | *-0.023* | *9.7%* | *0.002* | *-1.3%* |
| Duration of any eating disorder (ref: less than 1 year) |  |  |  |  |  |  |  |  |
| 1-3 years | 0.006 | -4.3% | -0.001 | -1.9% | -0.027 | 11.3% | -0.016 | 14.7% |
| Over 3 years | 0.011 | -8.2% | 0.011 | 15.6% | 0.011 | -4.7% | 0.008 | -7.7% |
| *Subtotal of duration of any eating disorder* | *0.017* | *-12.5%* | *0.010* | *13.7%* | *-0.016* | *6.6%* | *-0.008* | *7.0%* |
| **Total contribution of need variables** | **0.029** | **-22.1%** | **-0.004** | **-4.2%** | **-0.042** | **17.4%** | **-0.005** | **4.7%** |
| **Non-need variables** |  |  |  |  |  |  |  |  |
| Australia born | 0.000 | 0.3% | 0.002 | 2.7% | -0.003 | 1.1% | -0.003 | 2.9% |
| Marital status (ref: never married) |  |  | 0.003 | 4.3% |  |  |  |  |
| married or de-facto | 0.000 | -0.1% | 0.001 | 1.6% | 0.000 | -0.1% | -0.001 | 1.3% |
| widowed/divorced/separated | -0.003 | 2.0% | -0.003 | -4.0% | -0.001 | 0.5% | 0.000 | 0.1% |
| *Subtotal of marital status* | *-0.003* | *1.9%* | *-0.002* | *-2.4%* | *-0.001* | *0.4%* | *-0.001* | *1.4%* |
| Remoteness (Ref: major city) |  |  |  |  |  |  |  |  |
| Regional or remote | *-0.104* | *78.3%* | *-0.003* | *-4.0%* | *-0.009* | *3.6%* | *0.027* | *-25.1%* |
| Socioeconomic status  (SEIFA) (Ref: Decile 1: Most disadvantaged) |  |  |  |  |  |  |  |  |
| Decile 2 | -0.003 | 2.3% | -0.009 | -12.1% | -0.011 | 4.8% | -0.009 | 8.6% |
| Decile 3 | -0.003 | 2.0% | -0.016 | -22.1% | -0.016 | 6.9% | 0.005 | -5.0% |
| Decile 4 | -0.046 | 34.7% | -0.029 | -40.9% | -0.015 | 6.4% | 0.000 | 0.2% |
| Decile 5 | -0.006 | 4.3% | -0.008 | -10.6% | -0.011 | 4.6% | -0.002 | 1.4% |
| Decile 6 | -0.003 | 2.0% | 0.000 | 0.7% | -0.004 | 1.6% | -0.001 | 0.6% |
| Decile 7 | 0.003 | -2.2% | 0.011 | 15.1% | 0.000 | 0.0% | -0.003 | 2.5% |
| Decile 8 | 0.007 | -5.6% | 0.024 | 34.1% | 0.000 | 0.0% | -0.004 | 3.3% |
| Decile 9 | 0.011 | -8.6% | 0.036 | 50.5% | -0.037 | 15.7% | -0.038 | 34.7% |
| Decile 10 (Least disadvantaged) | -0.012 | 9.1% | 0.060 | 84.8% | -0.078 | 33.2% | -0.076 | 69.9% |
| *Subtotal of socioeconomic status* | *-0.052* | *38.0%* | *0.069* | *99.5%* | *-0.172* | *73.2%* | *-0.128* | *116.2%* |
| **Total contribution of non-need variables** | **-0.159** | **118.5%** | **0.066** | **95.8%** | **-0.185** | **78.3%** | **-0.105** | **95.4%** |
| Residual | -0.002 | 1.5% | 0.009 | 12.7% | -0.009 | 3.8% | 0.001 | -0.9% |

Notes: CCI =Contribution to the concentration index, contr.= contribution, cond.=conditional; residuals refer to unexplained components after accounting for the effects of the explanatory variables included in the model; contribution of need variables, non-need variables and residual factor sum to 100%. Please note that percentage may not total to 100% due to rounding in some cases.

## Supplementary Table 8: Decomposition analysis of eating disorder-related emergency department visits in 2020

|  | **Probability of visit** | | **Total number of visits** | |
| --- | --- | --- | --- | --- |
|  | CCI | % contr. | CCI | % contr. |
| **Need variables** |  |  |  |  |
| Female | 0.001 | -2.1% | 0.001 | -1.0% |
| Age (Ref: less than 15 years) |  |  |  |  |
| 15-24 years | 0.000 | 0.1% | 0.000 | 0.2% |
| 25-34 years | -0.001 | 3.2% | -0.003 | 2.9% |
| 35-44 years | 0.000 | -0.2% | 0.000 | -0.2% |
| 45 years and above | 0.001 | -2.6% | 0.003 | -2.9% |
| *Subtotal of age* | *0.000* | *0.5%* | *0.000* | *0.0%* |
| Comorbidities (Ref: No common psychiatric or medical comorbidity) |  |  |  |  |
| One or more common psychiatric comorbidity | -0.001 | 1.3% | 0.000 | -0.4% |
| One or more common medical comorbidity | 0.000 | 0.5% | 0.000 | 0.5% |
| One or more common psychiatric and medical comorbidity | 0.006 | -15.4% | 0.016 | -18.0% |
| *Subtotal of comorbidity* | *0.005* | *-13.6%* | *0.016* | *-17.9%* |
| Duration of any eating disorder (ref: less than 1 year) |  |  |  |  |
| 1-3 years | 0.009 | -21.2% | 0.011 | -11.9% |
| Over 3 years | -0.003 | 8.2% | -0.001 | 1.6% |
| *Subtotal of duration of any eating disorder* | *0.006* | *-13.0%* | *0.010* | *-10.3%* |
| **Total contribution of need variables** | **0.012** | **-28.2%** | **0.027** | **-29.2%** |
| **Non-need variables** |  |  |  |  |
| Australia born | 0.001 | -2.8% | 0.001 | -1.4% |
| Marital status (ref: never married) |  |  |  |  |
| married or de-facto | 0.002 | -5.7% | 0.002 | -2.5% |
| widowed/divorced/separated | 0.001 | -2.9% | 0.001 | -1.7% |
| *Subtotal of marital status* | *0.003* | *-8.6%* | *0.003* | *-4.2%* |
| Remoteness (Ref: major city) |  |  |  |  |
| Regional or remote | *-0.004* | *9.8%* | *-0.004* | *4.4%* |
| Socioeconomic status  (SEIFA) (Ref: Decile 1: Most disadvantaged) |  |  |  |  |
| Decile 2 | 0.001 | -2.7% | 0.000 | 0.0% |
| Decile 3 | 0.008 | -19.6% | 0.009 | -10.3% |
| Decile 4 | 0.000 | 0.3% | -0.008 | 9.0% |
| Decile 5 | -0.014 | 33.1% | -0.038 | 43.1% |
| Decile 6 | 0.001 | -3.5% | 0.001 | -0.7% |
| Decile 7 | -0.001 | 2.6% | 0.001 | -1.5% |
| Decile 8 | -0.004 | 9.1% | -0.008 | 9.0% |
| Decile 9 | -0.025 | 60.3% | -0.036 | 40.4% |
| Decile 10 (Least disadvantaged) | -0.021 | 50.2% | -0.037 | 41.2% |
| *Subtotal of socioeconomic status* | *-0.055* | *129.8%* | *-0.116* | *130.2%* |
| **Total contribution of non-need variables** | **-0.055** | **128.2%** | **-0.116** | **129.0%** |
| Residual | 0.001 | -2.4% | 0.000 | 0.0% |

Notes: CCI =Contribution to the concentration index, contr.= contribution, cond.=conditional; residuals refer to unexplained components after accounting for the effects of the explanatory variables included in the model; contribution of need variables, non-need variables and residual factor sum to 100%. Please note that percentage may not total to 100% due to rounding in some cases.

# Supplementary Figure

## Supplementary Figure 1: Distribution of study sample by SEIFA deciles
